# Urine-derived stem cells for potential use in bladder repair

**DOI:** 10.1186/scrt458

**Published:** 2014-05-28

**Authors:** Danian Qin, Ting Long, Junhong Deng, Yuanyuan Zhang

**Affiliations:** 1 Department of Physiology, Shantou University, Shantou, Guangdong 515041, China; 2 Department of Urology, Guangzhou First people’s Hospital, Guangzhou, Guangdong 510180, China; 3 Wake Forest University institute for Regenerative Medicine, Winston-Salem, NC 27106, USA

## Abstract

Engineered bladder tissues, created with autologous bladder cells seeded on biodegradable scaffolds, are being developed for use in patients who need cystoplasty. However, in individuals with organ damage from congenital disorders, infection, irradiation, or cancer, abnormal cells obtained by biopsy from the compromised tissue could potentially contaminate the engineered tissue. Thus, an alternative cell source for construction of the neo-organ would be useful. Although other types of stem cells have been investigated, autologous mesenchymal stem cells (MSCs) are most suitable to use in bladder regeneration. These cells are often used as a cell source for bladder repair in three ways - secreting paracrine factors, recruiting resident cells, and trans-differentiation, inducing MSCs to differentiate into bladder smooth muscle cells and urothelial cells. Adult stem cell populations have been demonstrated in bone marrow, fat, muscle, hair follicles, and amniotic fluid. These cells remain an area of intense study, as their potential for therapy may be applicable to bladder disorders. Recently, we have found stem cells in the urine and the cells are highly expandable, and have self-renewal capacity and paracrine properties. As a novel cell source, urine-derived stem cells (USCs) provide advantages for cell therapy and tissue engineering applications in bladder tissue repair because they originate from the urinary tract system. Importantly, USCs can be obtained via a noninvasive, simple, and low-cost approach and induced with high efficiency to differentiate into bladder cells.

## Introduction

Stem cell-based therapy for bladder repair is most relevant to congenital bladder conditions (for example, bladder exstrophy) or conditions such as radiation damage, infection, interstitial cystitis, neuropathic small bladder disease, and bladder cancer. Chronic bladder diseases cause reduced contractility and compliance, form heavy scar tissue, and significantly reduce bladder volume (end-stage bladder disease). To treat invasive malignancies or end-stage bladder diseases, a partial or total cystectomy is often used, followed by the creation of a neo-bladder or a continent urinary reservoir with an intestinal segment or gastric flap [1] to restore bladder function and increase its volume. However, using bowel tissue for this purpose commonly causes complications, such as excess mucus secretion, urinary tract infection, stone formation, and, most importantly, increased risk for malignancy, particularly adenocarcinoma, because of histological changes in the intestinal mucosa after long-term exposure to urine. Recent studies showed that all children with neurogenic bladder disease are at increased risk of bladder cancer regardless of exposure to intestine [2]. Therefore, new clinical and surgical techniques are needed to allow these patients to live healthier and more normal lives.

Bladder reconstruction with tissue engineering technology is possible through the use of normal autologous bladder cells seeded on biodegradable scaffolds [3]. However, in patients with end-stage bladder diseases or muscle-invasive bladder cancer, healthy autologous bladder cells might not be available. Concomitant development of a healthy, cancer-free stem cell source and an optimal three-dimensional nano-fibrous polymer scaffold are promising developments for use in patients who require cystoplasty.

Stem cells have shown potential as a therapeutic strategy for various tissue repairs, including of urinary bladder. Multiple types of cells have been used in preclinical animal models to repair or regenerate bladder tissue, employing either trans-differentiation or paracrine effects to stimulate endogenous cells participating in tissue regeneration. These stem cells include pluripotent stem cells such as embryonic stem cells (ESCs), induced pluripotent stem cells (iPSCs) [4], multi-potent mesenchymal stem cells (MSCs), bone marrow-derived mesenchymal stromal cells (BMSC) [5-9], adipose-derived stem cells [10], hair follicle stem cells [11,12], umbilical MSCs [13], urothelial stem cells [14] and, most recently, urine-derived stem cells (USCs) [15,16].

ESCs or iPSCs are naturally programmed to divide continuously and remain undifferentiated. Although these cells can give rise to ectodermal, mesodermal, or endodermal cell lineages, a significant risk of teratoma exists. Any undifferentiated ESCs or iPSCs placed in the body might continue to divide in an uncontrolled manner, forming tumors. In addition, it is time consuming (4 months) to derive and characterize iPSCs from an individual. Furthermore, low efficiency of cell differentiation, genetic abnormalities, and high cost prohibit clinical applicability. Even so, a few studies with ESCs or iPSCs for bladder tissue engineering have been reported. Frimberger and colleagues [17] reported that human embryoid body-derived stem cells showed improved migration in the presence of mature human bladder smooth muscle cells (SMCs) and urothelial cells (UCs). In addition, Moad and colleagues [4] reported the generation of human iPSCs derived from normal, ageing, human urinary tract tissue. These iPSCs were more efficient than skin-derived iPSCs in undergoing bladder differentiation as shown by expression of urothelial-specific markers (uroplakins, claudins, and cytokeratin) and stromal smooth muscle markers (alpha-smooth-muscle actin, calponin, and desmin), indicating the importance of organ-specific iPSCs for tissue-specific studies. Immobilized cell lines are not suitable for bladder regeneration due to safety concerns. Therefore, multi-potent adult stem cells are currently used in bladder repair and reconstruction.

## Mesenchymal stem cells for bladder repair

To be used successfully in therapies, MSCs must be directed to differentiate into the desired type of tissue. Three types of bladder cells, SMCs, UCs, and endothelial cells, are required for bladder regeneration [5-7,18-24]. Via trans-differentiation, MSCs can give rise to all three types in the bladder. In addition, MSCs possess paracrine effects, with anigogenic, anti-apoptosis, anti-fibrosis, anti-inflammatory properties [5-7]. BMSCs promote angiogenesis and increase cell viability of implanted UCs when both BMSCs and UCs seeded on biomaterial were transplanted *in vivo*[25]. Hypoxic stress increases generation of several of these cytokines and growth factors [26,27]. Thus, MSCs can recruit resident stem cells participating in tissue repair. Furthermore, MSCs purportedly exhibit low immunogenicity, allowing allogeneic applications [28-30].

MSCs have several advantages for tissue repair [18-24]: (i) they do not induce teratoma or malignant tumors; (ii) they can generate a large amount of cells within 4 weeks; (iii) they are highly efficient in giving rise to functional bladder cells, such as SMCs; (iv) they secrete paracrine factors that allow stem cells to be tolerated by the host’s immune system; and (v) their use avoids general ethical concerns that accompany use of other types of stem cells.

Although BMSCs or adipose-derived stem cells are the most commonly used MSCs, they have some limitations, such as low differentiation capacity (<5%) of UCs (endodermal lineage), short lifespan *in vitro* (<10 passages in BMSCs), and they require invasive collection procedures [31-34]. Thus, the ideal stem cell sources for bladder repair would: (i) be able to differentiate into functional UCs, endothelial cells, and peripheral neurocytes with high efficiency (these promote bladder contractility and compliance ability, and restore histological structures with innate vasculature and innervation); (ii) allow collection via a non-invasive, simple, safe, and low-cost method; (iii) have universal or ‘off the shelf’ availability; and (iv) generate tissue-specific or organ-specific stem cells from the urinary tract system. Currently, it is unknown whether such a ‘perfect’ stem cell exists. We do know, however, that certain cell types are more favorable than others.

## Urine-derived stem cells as a novel cell source

**Figure 1:**
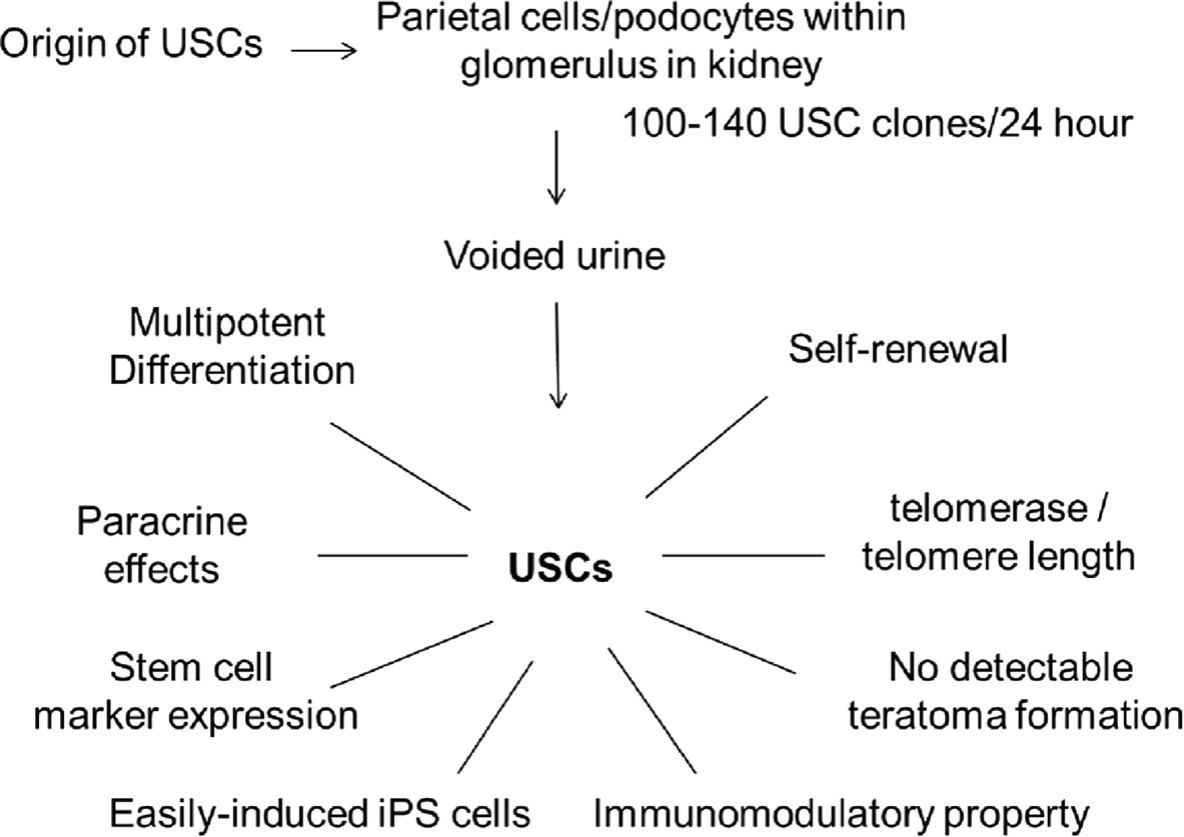
**Schematic illustration of the origin and biological characteristics of urine-derived stem cells (USCs).** USCs, a subpopulation of cells isolated from urine, possess biological characteristics similar to mesenchymal stem cells.

Although stem cells are a very small cell population, they play an important role in replacing aged, injured, and diseased cells and promoting tissue regeneration from organs where they originate. We recently found that a subpopulation of cells isolated from urine possess biological characteristics similar to MSCs; that is, clonogenicity, cell growth patterns, expansion capacity [15,35], cell surface marker expression profiles [15], multipotent differentiation capacity [16,36-40], pro-angiogenic paracrine effects [41,42], immunomodulatory properties [43] and easily induced iPSCs [44]. Thus, we have termed these cells 'urine-derived stem cells' or USCs (Figure 1). USCs consistently expressed MSC/pericyte markers and some key cell surface markers, but not hematopoietic stem cell markers (except for MHC-1), endothelial cell markers (CD31), or human leukocyte antigen (locus) DR (HLA-DR). Compared to other MSCs, USCs have several advantages: (i) they can be collected using a simple, safe, low-cost and non-invasive procedure; (ii) they display telomerase activity so that they are able to generate more cells; and (iii) they differentiate into SMCs, UCs and endothelial cells with high efficiency (Table 1).

**Table 1:** Comparison of various stem cell types used for bladder repair

**Cell type/parameters**	**BMSCs **[5-9,19,45-50]	**ASCs **[51,52]	**USCs **[15,16,35,36,38-40,53-55]	**ESC/iPSCs **[56]	**Bladder SMCs and UCs**
Self-renewal and expansion capability	Limited, PD ~30		High, PD 60-70	Very high, PD >200	Limited, PD <30
Multi-lineage differentiation capability	Multipotent, but mainly limited within mesodermal cell lineages [5-9]	Similar to BMSCs [52,54]	Multipotent differentiation potential [15,16,35,36]	Pluri-potent (can form all lineages of the body) [56]	None
Urothelial and endothelial differentiation capability	Low (<10%)	Low (10%)	High (60-85%) [39-41]	Low	
Telomerase activity (TA)/telomere length	Cannot be detected	Cannot be detected	Up to 75% USC clones possess TA and relatively long telomeres	Possess TA and long telomeres	None
Harvesting approach	Invasive	Invasive	Non-invasive, simple, cost-low, safe [56]	Invasive to harvest somatic cells for iPSCs	Invasive
Pure stem cell isolation	Difficult	Difficult	Very easy	Easy	None
Number of stem cells harvested	1 MSC/10^4^ bone marrow stromal cells in new borns, 1 MSC/10^6^[19]		100-140 USC clones/24 hour urine in adult [55,57]		Unknown
Angiogenic trophic factors	Yes	Yes	Yes	Unknown	Moderate
Immuno-modulatory properties	Yes	Yes	Yes	Unknown	Unknown
Rejection after implanted *in vivo*	No rejection reaction as allogenous or even xenogenous cells (for example, human BMSCs, USCs) implanted in rodent, rabbit, or canine models			Likely to be rejected	No rejection as autogenous cells
Oncogenic potential	No	No	No	Yes	None
Clinic trial utility	Potential	Potential	Potential	Safety concern	Yes

ASC, adipose-derived stem cell; BMSC, bone marrow-derived mesenchymal stromal cell; ESC, embryonic stem cell; iPSC, induced pluripotent stem cell; MSC, mesenchymal stem cell; PD, population doubling; SMC, smooth muscle cell; UC, urothelial cell; USC, urine-derived stem cell.

### Proliferation capacity of urine-derived stem cells

USCs can be obtained from voided urine and can generate a large number of cells from a single clone [37,38]. These cells possess highly proliferative capacity because they maintain higher telomerase activity and longer telomere length compared to BMSCs. Up to 75% of USCs collected from middle-aged individuals expressed telomerase activity (USCs-TA^+^) and retained long telomere length [58], but USCs-TA^+^ decline to 50 to 60% of the USCs in people aged 50 years old or older. USCs-TA^+^ can be maintained for up to 20 passages with 67 population doublings, indicating that a single USC can generate up to 2^67^ cells within 14 weeks. In contrast, USCs-TA^−^ grow for only 8 to 10 passages with 34 population doublings. Importantly, either USCs-TA^+^ or USCs-TA^−^ display normal karyotypes in culture medium even after several passages. They did not form teratomas 3 months after renal subcapsular cell implantation [58]. We can now obtain 100 to 140 USC clones/24 h urine from each individual [35]. About 1.4 × 10^9^ cells are needed for potential use in bladder reconstruction with cell-seeded technology [3]. Thus, two urine samples containing 20 to 30 USC clones in 400 ml can provide ample cells (1.5 × 10^9^ USCs at passage 4) within 4 to 5 weeks to be used in cell-based therapy for bladder repair.

### Multipotent differentiation potential of urine-derived stem cells

Our data demonstrated that USCs are capable of osteogenic, chondrogenic and adipogenic myogenic, neurogenic and endothelial differentiation [15]. After being induced in the appropriate condition *in vitro*, each type of differentiated USC expressed specific markers at the gene, protein, and cellular levels. Following implantation *in vivo*, induced USCs can form functional bone, cartilage, fat, muscle, endothelium, and urothelium tissue [15]. However, signaling pathways involved in USCs differentiation and proliferation need further investigation.

### Urine-derived stem cell differentiation into bladder cells

For bladder tissue engineering, urothelial cells are needed for creating bladder mucosa, smooth muscle cells for building up bladder wall, and endothelial cells for forming blood vessels. However, a challenge in urological tissue regeneration is generating urothelial cells from MSC-derived cells. Although BMSCs, the most commonly used MSC source, can efficiently differentiate into SMCs, only 5% of BMSCs can give rise to the cells expressing urothelial markers *in vitro*[40]. One of the most likely reasons for this is that true stem cells in bone marrow stromal cells are very rare, depending on donor age (1/10^4^ cells in newborns, but 1/10^6^ in older individuals). Furthermore, it is very difficult to isolate stem cells from the large amount of somatic cells. Anumanthan and colleagues [45] reported that use of embryonic rat bladder mesenchymal cells co-implanted with allogeneic rat BMSCs induced bladder tissue structure with cells expressing urothelial and muscle markers. As well as urothelial differentiation of MSCs, Nagele and colleagues [59] reported that human urothelial cells can be harvested from bladder washings and primary cultures of these were successfully established from half the bladder wash samples. The cultured cells formed multilayered urothelial sheets for potential use in urinary tract tissue reconstruction. Recently, Drewa and colleagues [12] found that hair follicle stem cells from rat whisker hair follicles differentiated into cells expressing epithelial cell markers (cytokeratin 7, cytokeratin 8, cytokeratin 18) during culture in UC-conditioned medium for 2 weeks *in vitro*.

Using the same inductive medium as in the BMSC study [6], we found that 60 to 70% of USCs differentiated into cells expressing UC-specific genes (uroplakin-Ia/III) and protein markers, and had urothelial barrier function and tight junction ultrastructures. Urothelial differentiated USCs also expressed the genes encoding ZO-1, E-cadherin, and cingulin as well as the protein products (associated with tight junctions) in a dose- and time-dependent manner. The barrier function of induced USCs reached the mature function of UCs isolated from bladder tissue 14 days after induction, significantly higher than for non-induced USCs, indicating that the USCs possessed stem cell plasticity.

USCs can efficiently give rise to functional cells of the SMC lineage. Smooth muscle differentiated USCs expressed a-SM actin and calponin, desmin and myosin, and smoothelin at both the gene and protein levels [15,16]. The mRNA and protein levels of these markers increased significantly with time in differentiation media. Functional studies demonstrated that these SMCs have contractile properties *in vitro*. Myogenic differentiated USCs formed multiple layers of SMCs beneath UC layers when subcutaneously implanted in a nude mouse model [15,38]. The SMCs stained positively for a-SM actin, desmin, and myosin. Scaffolds containing urothelial differentiated USCs generated stratified layers *in vivo* and stained positive for uroplakin-Ia and uroplakin-III (urothelial markers) [14,38].

We found that USCs differentiate into cells of the endothelial lineage when grown in endothelial differentiation medium containing 2 ng/ml vascular endothelial growth factor (VEGF) for 12 days [15]. *In vitro* 'vessel forming' was displayed 18 hours after differentiated USCs (5 × 10^3^ cells) were seeded onto Matrigel. The differentiated cells began to express the specific gene and protein markers of endothelial cells (CD31, vWF, KDR, FLT-1, FLT-1, eNOS and VE-cadherin). Induced USCs demonstrated intense immunofluorescent staining for these markers compared to non-differentiated USCs. Importantly, USCs can be efficiently differentiated into endothelial cells with barrier function. Neovessel formation occurred after induced USCs were subcutaneously implanted in an athymic mouse model [40,41].

### Immunoregulatory properties of urine-derived stem cells

Regulatory T cells play an important role in induction of peripheral tolerance, inhibition of pro-inflammatory immune responses, and decreased immune reactions. We recently demonstrated that USCs can impart profound immunomodulatory effects, inhibit proliferation of peripheral blood mononuclear cells (PBMNCs; T and B cells), and secrete IL-6 and IL-8 [43]. PBMNCs proliferated when mixed with other cells due to immune stimulation. The PBMNC concentration in USC wells was much less than that in BMSC culture wells. Bromodeoxyuridine colorimetric enzyme-linked immunosorbent assays showed there was less bromodeoxyuridine label in the mixed USC and PBMNC culture wells compared to the BMSC culture wells. CD80 and CD86 expressed on the surface of antigen‒presenting cells interact with cytotoxic T lymphocyte antigen‒4 expressed on activated T cells and mediate critical T-cell inhibitory signals. Flow cytometry showed that 3.35% of the BMSCs were positive for CD80 (versus 1.05% of USCs), and 1.3% of the BMSCs were positive for CD86 (versus 0.55% of USCs). Human cytokine release arrays showed that IL-6 and IL-8 concentrations were elevated after stimulation by PBMNCs in USC supernatant to a greater degree than in BMSC supernatant. IL-6 and IL-8 might be the main immunomodulatory cytokines to target in future studies aimed at preventing and treating diabetic bladder tissue lesions, other immune system disorders, or rejection of transplanted organs.

### Origin of urine-derived stem cells

USCs isolated from urine obtained from the upper urinary tract are similar to voided USCs in morphology, cell phenotype, growth pattern, and differentiation capacity [36]. We found strong evidence that the voided USCs originate from the kidney, because cells obtained from women who had received transplanted kidneys from male donors contained the Y chromosome and expressed normal renal cell markers (PAX2 and PAX8), podocytes and parietal cells (which populate the glomerulus in kidney [60-68]), and specific gene and protein markers (synaptopodin and podocin). USCs also expressed CD146 at a rate similar to that expressed in parietal cells and podocytes in glomerulus, while bladder and ureter UCs and SMCs did not, indicating that USCs are likely transitional cells at the parietal cell/podocyte interface originating from renal tissue. Recently, parietal cells have been considered as stem cells in the glomeruli, displaying self-renewal properties and the potential to give rise to podocytes and proximal tubular cells [60-68]. Parietal cells are commonly obtained from kidney tissue biopsies, but the isolation of pure parietal cells is difficult [63-68].

In chronic bladder diseases, USCs might be a good cell source for bladder tissue regeneration because the cells from the upper urinary tract are normal. For treatment of end-stage bladder diseases or muscle-invasive bladder cancer, using bladder tissue created with USCs would be superior to bladder reconstruction using intestinal segments.

### Impact of angiogenic growth factors on urine-derived stem cell ingrowth and differentiation *in vivo*

USCs can secrete angiogenic growth factors and cytokines, but require a favorable microenvironment to do so. We demonstrated that use of genetically modified stem cells via transfection of the VEGF gene significantly promoted myogenic differentiation of USCs and induced angiogenesis and innervation [69]. However, virally delivered VEGF caused several side effects in our animal model, including hyperemia, hemorrhage, and even death [53]. Thus, a safer approach is needed for stem cell therapy to increase angiogenesis and promote muscle regeneration. Adding exogenous angiogenic factors into biodegradable polymers as delivery vehicles can be beneficial to promote regeneration and tissue healing [57]. Alginate is one of the most commonly used natural hydrogels as an aqueous drug carrier for encapsulation because of its mild gelling conditions and tunable microsphere characteristics. Alginate microbeads also resist protein adsorption, making them attractive for *in vivo* studies [70]. Alginate microbeads deliver molecules in a controlled fashion, which can stably release active fibroblast growth factor (FGF)-1 for at least 3 weeks *in vitro*. This sustained release of FGF-1 promoted neovascularization *in vivo* without any side effects [71-73]. More recently, we found that a combination of growth factors (VEGF, insulin-like growth factor-1, FGF-1, platelet-derived growth factor, hepatocyte growth factor and nerve growth factor) released locally from alginate microbeads induced USCs to differentiate into a myogenic lineage, enhanced revascularization and innervation, and stimulated resident cell growth *in vivo*[53]. In addition, when cultured on three-dimensional biomaterial, stem cells had significantly enhanced cell viability, proliferation, and differentiation *in vitro*, and promoted tissue formation *in vivo*, compared to cells cultured on two-dimensional plates [74].

### Biomaterials for bladder tissue regeneration

Two types of biodegradable scaffolds are commonly used in cell-seeded tissue engineering for bladder reconstruction: natural collagen materials (that is, bladder submucosa [75] or small intestine submucosa [39,76] and collagen type I matrix [77]); and synthetic polymers such as polyglycolic acid and poly(lactic-co-glycolic acid) [3,78], biocarbon [79], poly-L-lactic acid (PLLA) [8,9] and bacterial cellulose polymer [38]. Most degradable biomaterials promote cellular interaction and tissue development, and possess adequate mechanical and physical properties. However, natural collagen scaffolds cannot maintain a robust physical structure in an *in vivo* environment when used in total or subtotal bladder replacement, resulting in graft collapse, contraction, formation of fibrosis, and shrinkage of the new bladder, with resultant decreased bladder capacity [22]. A biomaterial that retains a hollow structure, and has anti-fibrosis properties and a three-dimensional porous microstructure for graft cell seeding would be highly desirable for creating a viable tissue-engineered bladder.

An ideal biological material for urethral tissue engineering would have high porosity and appropriate pore sizes to allow cell attachment, migration and penetration into the matrix after seeding, and it should be able to induce angiogenesis, be biodegradable, histocompatible, and have the least xenogenous antigens retained within the matrix for minimum inflammatory potential. The synthetic material nanofibrous PLLA appears to meet these criteria, as it possesses a three-dimensional, highly porous structure (50 to 200 μM), but has adequate tensile strength to maintain the shape of an engineered organ *in vivo*. The highly interconnected pore structure of the nanofibrous PLLA scaffolds is an excellent environment for cell growth and angiogenesis [80,81]. This allows abundant cell loading onto the scaffold, and induces microvessel network formation, thereby promoting *in vivo* tissue regeneration and wound healing [82-85]. It also allows the host’s cells to participate in tissue remodeling processes by encouraging infiltration or migration into the matrix from the wound edges. Because of these properties, PLLA has been used in a number of tissue engineering studies [9,81,83,84,86-119], including on blood vessels [86,88]. Importantly, the nanofibrous PLLA scaffolds degrade significantly faster than control PLLA scaffolds because of their drastically higher surface area and fiber size [106], making them suitable for bladder tissue regeneration.

## Debated hypotheses

Several assumptions are still controversial in bladder tissue regeneration research. First, trans-differentiation and paracrine effects are both critical in regeneration of various tissues. Although most studies have monitored the survival rate of implanted cells, cytokines and growth factors secreted from stem cells might play an important role in bladder repair [120].

Second, the need for addition of growth factors to induce angiogenesis is still debated. However, accumulating evidence shows that extrogenous growth factors improve blood support and tissue repair [121,122], especially in local tissues with poor blood supply or when large amounts of bladder tissue are being replaced. Growth factors may not be required where the local tissue possesses a rich blood supply or when only a small amount of bladder tissue needs replacement [22].

Third, should undifferentiated or differentiated MSCs be used in bladder repair [19]? Undifferentiated stem cells can secrete more paracrine factors than differentiated stem cells, but differentiated cells might possess more potential to replace dysfunctional somatic cells. Therefore, a 1:1 ratio of undifferentiated and differentiated cells might be optimal for bladder regeneration.

Fourth, both layered co-culture and sandwich co-culture seeding techniques are used [20]. The former allows the interaction of epithelial-stromal cells, whereas the latter provides a histologic structure more similar to the normal structure, thereby preventing SMC loss during surgical procedures. In addition, expression of the protein signal sonic hedgehog in urothelial basal cells boosts and provokes increased stromal expression of Wnt protein signals, which in turn stimulate the proliferation of both urothelial and stromal cells.

## Future directions

Using stem cells more efficiently for bladder regeneration requires improving angiogenesis, inducing innervation, and developing more suitable biomaterials in the next few years. High short-term cell retention and long-term engraftment after cell delivery allow more successful bladder tissue repair during regeneration. Cell retention within 24 hours of delivery in the bladder is relatively high (regardless of the cell type or scaffolds) when SMCs are seeded on the serosal side. However, UCs seeded on the luminal side of the scaffold are often lost during surgery procedures, washed out via the urine, or mechanically ejected via the urethral catheter. In addition, successfully retained cells start to die within the first week, most probably due to ischemia, inflammation, or apoptosis due to detachment from the extracellular matrix. Therefore, it is extremely important to increase viability of implanted stem cells early after cell transplantation. Several methods might help reach this goal: (i) using biomaterials with a porous micro-structure that might protect cell retention within the scaffold; (ii) keeping the cell-seeding scaffold construct wet in the culture media, and avoid drying it out during surgery; (iii) inducing angiogenesis or capillary network formation early in implantation with angiogenic growth factors released from microbeads or binding scaffolds in the site or using hypoxia as a pretreatment for implanted cells; and (iv) promoting revascularization (artery-capillary-venous system) at the mid or late stage after the implantation with biologically safe physical stimulation, including lower-frequency electrical stimulation or low-intensity ultrasound. These methods could extend the lifespan of implanted cells *in vivo* to provide better tissue repair with long-term release of paracrine factors and trans-differentiation, anti-fibroblast formation, and anti-inflammatory and anti-apoptotic effects of MSCs. In addition, innervation is critical to create a functional bladder. Stimulating peripheral nerve growth into neo bladder tissue might be more efficacious than attempting to create neurogenic differentiation of MSCs.

## Conclusion

Use of MSCs, which possess an excellent safety profile, for bladder tissue regeneration is highly feasible. Pre-clinical outcomes have been generally positive in restoring bladder contractility and volume in the partial (40%) cystoplasty model. Autologous MSCs derived from patients would be a potential cell source for bladder repair. MSCs appear safe to use for urological tissue repair with no evidence of increased tumorigenesis after implantation. USCs possess MSC features, including self-renewal, multi-differentiation potential, and paracrine effects. As a novel cell source, USCs can be obtained via a non-invasive, simple, safe and low-cost approach, are highly expandable, give rise to bladder cells efficiently, and express telomerase activity but do not induce teratomas. Studies of implanted USCs *in vivo* will help to determine their impact on bladder tissue regeneration and monitor cell retention and engraftment over the longer term (beyond 3 months). Besides bladder tissue repair, USCs might also be a viable cell source for uretera or urethral tissue engineering and reconstruction, and for cell therapy in treatment of diabetic erectile dysfunction, vesicoureteral or anal reflux and other diseases.

## Note

This article is part of a thematic series on *Stem cells in genitourinary regeneration* edited by John Jackson. Other articles in the series can be found online at http://stemcellres.com/series/genitourinary

## Abbreviations

BMSC: Bone marrow-derived mesenchymal stromal cell; ESC: Embryonic stem cell; FGF: Fibroblast growth factor; IL: Interleukin; iPSC: Induced pluripotent stem cell; MSC: Mesenchymal stem cell; PBMNC: Peripheral blood mononuclear cell; PLLA: Poly-L-lactic acid; SMC: Smooth muscle cell; UC: Urothelial cell; USC: Urine-derived stem cell; VEGF: Vascular endothelial growth factor.

## Competing interests

The authors declare that they have no competing interests.

## Authors’ information

Danian Qin and Ting Long are first co-authors.
